# High-frequency direct somatic embryogenesis and plantlet regeneration from date palm immature inflorescences using picloram

**DOI:** 10.1186/s43141-021-00129-y

**Published:** 2021-02-18

**Authors:** Mona M. Hassan, Mai A. Allam, I. M. Shams El Din, Mervat H. Malhat, Rania A. Taha

**Affiliations:** 1 The Central Laboratory of Date Palm Researches and Development, Agriculture Research Center, Giza, Egypt; 2 Regional Development Centre of New Valley, Academy of Scientific Research and Technology, 101 Kasr El-Ainy St, Cairo, 11694 Egypt; 3 Plant Biotechnology Department, Genetic Engineering and Biotechnology Division, Centre of Excellence for Advanced Sciences) National Research Centre, 33 Elbohouth St., Dokki, Giza, 12622 Egypt; 4 Academy of Scientific Research and Technology, Regional Development Centers (RDC), 101 Kasr El-Ainy St., Cairo, 11694 Egypt; 5 Tissue Culture Laboratory of Agricultural Genetic Engineering Research Institute, Agriculture Research Center, Giza, Egypt; 6 Tissue Culture Technique Lab., Pomology Department, Agriculture and Biology Research Division and Central Laboratories Network, National Research Centre (NRC), 33 Elbohouth St., Dokki, Giza, 12622 Egypt

**Keywords:** Inflorescence, Somatic embryogenesis, *Phoenix dactylifera*, Picloram

## Abstract

**Background:**

Date palm (*Phoenix dactylifera* L.) is a traditional crop in arid and semi-arid areas. Its vegetative propagation can be achieved by offshoots, but possible number of offshoots in mother palm trees is limited. Micropropagation is a highly recommended strategy for obtaining date palm elite cultivars using shoot tip and immature inflorescences. In this study, micropropagation procedure using inflorescence explants of Medjool cv. is described. For culture initiation, explants from different spathe lengths were cultivated on Murashige and Skoog medium (MS) supplemented with picloram at 1.0 and 2.0 mg/l combined with 2iP at 0.5 mg/l alone and with both 2iP and BA at 0.25 mg/l for 24 weeks. The obtained direct globular embryos were transferred to maturation media with 0.1 mg/l picloram alone or combined with both 2iP and ABA separately and together for further development. Additionally, multiplication and rooting media were optimized by different cytokinins and auxins for high frequency of plantlet production. Acclimatization of *in vitro* plantlets was also investigated.

**Results:**

The highest percentage of globular embryo formation was noticed with explants isolated from spathe lengths ranging from 10 to 15 cm. Addition of BA to initiation media with picloram encouraged a significant effect on embryonic culture formation percentage. Incorporation of ABA and 2iP to maturation medium was an effective factor for individual or multiple embryo emergence. Acclimatization of *in vitro* plantlets having 3–4 roots was successfully accomplished. Irrigation with the full strength solution (MS) encouraged the highest growth vigor degree, leaf number/plant, leaf width, root number, and root thickness degree of *ex vitro* plants.

**Conclusion:**

This research provides an advanced regeneration system for large-scale production of date palm from immature inflorescences of Medjool cv. It opens up the prospects of using picloram with different growth regulators for rapid micropropagation of date palm.

## Background

Date palm, *Phoenix dactylifera* L., belongs to the Arecaceae family. It is considered to be one of the most important monocotyledonous fruit trees in arid and semi-arid regions [1]. Date palm is generally propagated by offshoots. However, date tree produces a limited number of offshoots over its life duration. This is due to offshoots being produced only during the vegetative development phase of the palm [2]. *In vitro* culture techniques have been used to overcome the shortage in traditional propagation method and considered as an alternative method for large-scale production of the date palm [3]. Micropropagation of date palm has been achieved using shoot tips, mature and immature inflorescences [4–6]. Immature inflorescence has become ideal explants that could be used to propagate rare and superior palms especially those that do not produce offshoots. Furthermore, it has many advantages over shoot tip explants: low contamination percentage, less browning degree at initiation stage, and short propagation duration; additionally, a large number of explants is available from one spathe [7–9].

*In vitro* propagation of date palm through inflorescence cultures has been reported recently by several researchers based on either somatic embryogenesis [5] or organogenesis [6, 10–12]. Somatic embryogenesis is considered a model for understanding the biochemical and physiological changes that occur during plant growth processes, in addition to being one of the components of biotechnological progress [13]. Somatic embryogenesis has tremendous potential for rapid large-scale plant production. This technology can be used for the large-scale propagation of date palm [14, 15]. For decades, somatic embryogenesis has been used for date palm micropropagation from apical shoot tips and lateral buds [16, 17], but recently, it has been accelerated from inflorescence [7].

Nutrient medium and exogenous application of plant growth regulators (PGRs) have a vital role in *in vitro* growth, differentiation, and plant regeneration of date palm [18]. Composition and relative concentration of PGRs determine both the ability of the explant to respond as well as the mode of the morphogenic reaction [19]. Furthermore, the incorporation of plant growth inhibitors into the culture medium at an appropriate concentration modifies plant structure in a typical manner [20]. The inclusion of certain gibberellin biosynthesis inhibitors such as ancymidole, paclobutrazole (PBZ), or uniconazole influenced positively the somatic embryogenesis and plant regeneration of several species [21]. PBZ inhibits gibberellin biosynthesis, stimulates accumulation of ABA and changes cytokinin concentrations. Moreover, abscisic acid (ABA) has been observed to influence morphogenesis in a number of plants by modifying the effects of other hormones—cytokinins, auxins, and also gibberellins [18].

To induce somatic embryogenesis in date palm, the auxin 2, 4-dichlorophenoxy acetic acid (2,4-D) has been widely used at the concentration of 100 mg/l [22]. However, it was reported that high concentrations of 2,4-D may induce somaclonal variation within regenerants [23]. Therefore, inducing somatic embryogenesis in date palm using other auxins at low concentrations would be of great interest. Abahmane [24] reported that dicamba and picloram (auxin congeners) have been used successfully in tissue culture of various species with no adverse effects on regenerated plants.

Picloram (4-Amino-3,5,6-trichloro-2-pyridinecarboxylic acid) has been scarcely used to induce somatic embryogenesis in date palm. Othmani et al. [25] used picloram at different concentrations to induce somatic embryogenesis from juvenile leaves of date palm cv. Boufeggous and reported that this auxin failed to produce embryogenic callus. Khierallah et al. [26] succeeded to produce embryogenic calli in date palm cv. Bream using 50 mg/l picloram. More recently, Mazri et al. [27] succeeded to induce somatic embryogenesis in cv. Najda using 10.87 mg/l picloram.

This study aims to optimize date palm production protocol of Medjool cv. through immature female inflorescence. Thus, the effects of different spathe lengths of inflorescence, different combinations of picloram with various plant growth regulators (PGRs) at initiation and maturation stages, and their impacts on growth behavior were investigated in terms of explant browning, swelling, fresh weight, individual embryos, multiple embryos, and vitrified embryos. The effect of cytokinin treatments on the multiplication and shoot formation of date palm was also studied. Moreover, estimation of growth measurements expressed as plant length, growth vigor, and leaf number after 24 weeks in greenhouse as affected by MS salt solution was discussed.

## Methods

This work was performed in the Biotechnology Laboratory, New Valley Regional Centre, Egypt, in the period of 2019 to 2020.

### Plant materials

**Fig. 1:**
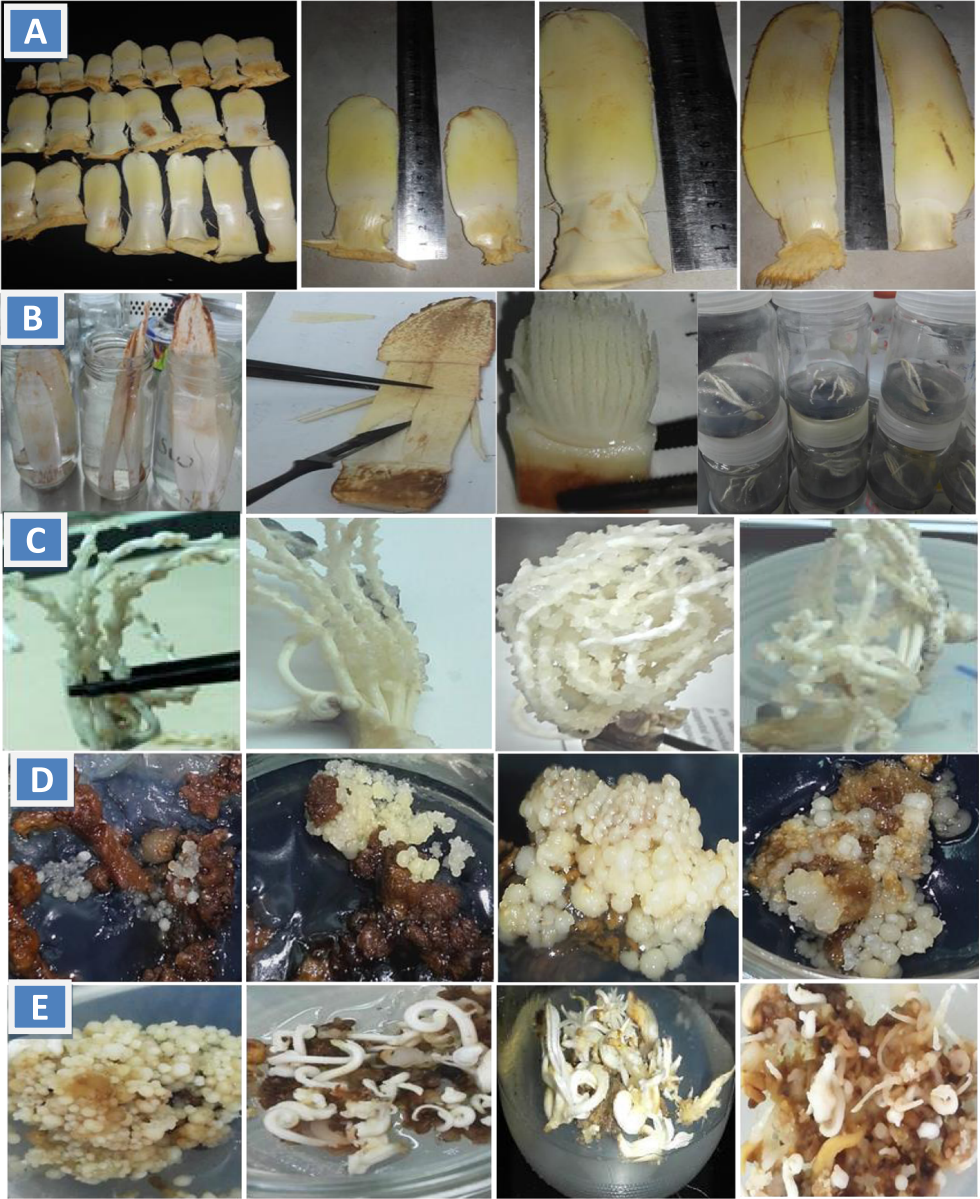
*In vitro* stages for Medjool direct embryogenesis. **a** Various spathe lengths. **b** Procedures from sterilization to initiation culture of spathes. **c** and **d** Various responses according to spathe lengths 3–5, 5–10, 10–15, and 15–20 cm from left to right, respectively; cultured on 2.0 picloram + 0.25 2iP + 0.25 BA mg/l; note the higher response of the third length in initiation stage (c) and globularization (d). **e** Different responses during maturation stage; from left to right: heaviest embryonic cultures, individual embryos, multiple embryos, and vitrified embryos

Immature inflorescences of Medjool cv. were excised from adult date palm female trees [1] grown at respected farm in Cairo-Alexandria road (the latitude is 30.033333 and the longitude is 31.233334), Egypt, on late January and used as explant materials in this investigation. Different spathe lengths were used: small (3–5 cm), intermediate (5–10 cm and 10–15 cm), and finally long (15–20 cm) (Fig. 1a).

### Surface sterilization and explant preparation

The spathes were immersed into fungicide solution (1.0 g/l Moncut) for 5 min followed by washing under tap water. A 10% sodium hypochlorite (NaOCl) solution with few drops of tween 20 was used as surfactant for 5 min with small spathes (3–5 cm) and for 10 min with other lengths. After sterilization, different spathes were rinsed three times with sterilized distilled water. The outer protective sheath was gently removed to avoid any damage of the spiklets inside. The spiklets were divided into pieces; each one contains 4–5 immature florets and cultured on nutrient medium (Fig. 1b).

### Initiation culture medium

Explants from different spathe lengths were initially cultured on initiation medium consisting of MS [28] supplemented with the following treatments:

1.0 mg/l picloram + 0.5 mg/l iso-pentenyl adenine (2iP), 1.0 mg/l picloram + 0.25 mg/l 2iP+ 0.25 mg/l benzyladenine (BA), 2.0 mg/l picloram + 0.5 mg/l 2iP and 2.0 mg/l picloram + 0.25 mg/l 2iP+ 0.25 mg/l BA.

All culture media were amended with 100 mg/l glutamine, 0.5 g/l activated charcoal, 2.0 g/l polyvinylpyrrolidone (PVP), and 30 g/l sucrose and solidified with 6 g/l agar. The pH was adjusted to 5.7–5.8 prior to addition of agar. Medium was dispensed into small jars (250 ml) at 35 ml per jar. Jars were capped with polypropylene closures and sterilized at 121 °C and 15 ibs\ins^2^ for 20 min. All cultured explants were incubated in a controlled growth room at 27 ± 2 °C under darkness (24 h) for 24 weeks (8-week intervals). Swelling degree of explants was recorded after 8 weeks, while percentage of response (number of explant show embryonic culture emergence/total cultured number ×100) and browning degree were recorded after 24 weeks.

### Maturation medium

Embryonic culture formed previously (0.5 g) were transferred to 3/4 MS medium consists of: 0.1 mg/l picloram, 0.1 mg/l picloram + 0.1 mg/l 2ip, 0.1 mg/l picloram + 0.1 ABA and 0.1 mg/l picloram + 0.1 mg/l 2ip +0.1 mg/l ABA. The control (medium without growth regulators) was also used. All cultures were maintained under darkness (24 h) for 12 weeks (8-week interval). After this period, culture fresh weight, individual, multiple, and vitrified embryo numbers per multiple embryo were estimated.

### Multiplication medium

Individual embryos from the previous medium were collected and cultured on half-strength MS medium supplemented with 0.1 mg/l NAA + 0.05 mg/l BA + 0.5 g/l activated charcoal (AC) and dispensed into small test tubes (2.5 × 15 cm) for 8 weeks (4-week intervals). After that, plantlets were transferred to larger test tubes (2.5 × 25 cm) to complete growth and development.

Otherwise, clusters of multiple embryos derived from maturation stage (each one contains 3–4 embryos) were cultivated on half-strength MS medium supplemented with 0.1 mg/l NAA+ 0.2 mg/l ABA alone (as control medium) or added with the following cytokinins: 0.25 mg/l 2ip, 0.25 mg/l BA, 0.25 mg/l kinetin and the combination of 0.1 mg/l 2ip + 0.1 mg/l kinetin+ 0.1 mg/l BA. All culture media were used with and without activated charcoal at 1.0 g/l and dispensed into glass jars (200 ml) at 30 ml/jar. Cultured jars were maintained for 3 recultures (3-week intervals) under low light intensity (500 lux). Numbers of embryos, shoots, and vitrified embryos/jar were estimated.

### Elongation medium

Clusters of developed shoot (4–5 shoots) which resulted from multiplication stage were transferred to liquid culture media to obtain healthy individual shoots for rooting stage. Liquid culture medium consists of half-strength MS medium added with 0.5 mg/l kinetin, 0.2 mg/l NAA, 0.2 mg/l paclobutrazol (Pbz), 40 g/l sucrose, and 2.0 mg/l Ca-pentothianate with 0.5 g/l AC and dispended into big jars (375 ml) at 25 ml/jar. All cultures were continued in liquid medium for 8 weeks (4-week interval) and incubated at 27 ± 2 °C with light intensity of 3000 lux.

### Rooting medium

Healthy individual shoots were cultured on solid MS salt medium supplemented with 0.2 mg/l NAA, 0.1 mg/l indole-3-butyric acid (IBA), 0.1 mg/l Pbz, 30 g/l sucrose, 2.0 mg/l glycine, 5.0 mg/l thiamine HCl, and 1.0 mg/l biotin solidified with 6.0 g/l agar. Rooting medium was dispensed on culture tubes (2.5 × 25 cm) at 25 ml/tube. Shoots were continued for 12 weeks (6-week interval) in test tube to form roots. Rooted shoots were transferred to half-strength liquid MS medium for an additional 6 weeks as pre-acclimatization stage. Plantlets on both solid and culture media were incubated at 27 ± 2 °C with light intensity of 4000 lux.

### Acclimatization process

Healthy rooted shoots (10–12 cm) with 2–3 leaves and 1–3 roots were selected and shifted to greenhouse. Rooted plantlets were gently removed from test tubes and then rinsed with tap water. Plantlets were immersed in 1% (w/v) fungicide solution (Moncut 25%) for 20 min. Thereafter, the plantlets were transplanted into plastic pots (5 × 18 cm) filled with peat: perlite mixture (2:1 v/v).The plantlets were kept at 27 °C—natural day light and high relative humidity (90–95%) using a cover of white transparent polyethylene sheets for 4–6 weeks. The polyethylene sheets were progressively removed to allow plant acclimatization under greenhouse conditions [29]. Acclimatized plants developed new roots in *ex vitro* conditions after 12 weeks. After that, plants having 2–3 leaves were irrigated twice a week with ¼, ½, and full MS inorganic salts for 24 weeks. Plant length (cm), leaf number, and root system were estimated visually according to Pottino [30] and recorded after 24 weeks.

### Statistical analysis

Data for swelling was collected after 8 weeks, while data for browning and embryonic culture were collected after three recultures with eight-week intervals. Scores were determined according to the rate of scaling by Pottino [30], which included the following: negative results = 1, below average = 2; average = 3, above average = 4, and excellent = 5. Treatments were arranged in complete randomized design; each treatment replicated three times; each replicate contained 5 jars, and each contained one explant. Means were compared according to the method described by Snedecor and Cochran [31].

## Results

###  Browning degree

Data in Table 1 showed the browning degree of explants removed from different lengths of spathes and cultured on initiation medium with different growth regulator supplements. It is clear that the lowest significant mean values of browning were recorded with intermediate lengths (5–10 and 10–15 cm) in respective order with significant difference. However, the highest significant mean values were noticed with smaller and longer lengths, respectively. Generally, initiation medium enriched with 2 mg/l picloram and either 2iP (at 0.5 mg/l) or both BA and 2iP (at 0.25 mg/l) achieved the lowest significant values of browning. Interaction revealed that explants taken out from intermediate lengths and cultured on MS added with 2.0 mg/l picloram and either 2ip or both 2iP and BA produced the lowest significant values of browning.

**Table 1:** Effect of spathe length and combination between picloram, 2iP, and BA on the browning degree of date palm explants after 24 weeks

Treatments (mg/l)	Spathe length				Mean
	3–5 cm	5–10cm	10–15cm	15–20 cm	
1.0 Picloram + 0.5 2iP	4.44 a	3.22 c	3.88 b	4.22 ab	3.94 A
1.0 Picloram + 0.25 2iP + 0.25 BA	3.77 b	3.00 cd	3.22 c	3.77 b	3.44 B
2.0 Picloram + 0.5 2iP	3.11 cd	2.66 de	2.77 cd	3.20 c	2.94 C
2.0 Picloram + 0.25 2iP + 0.25 BA	2.88 cd	2.22 ef	2.16 f	2.77 cd	2.51 D
Mean	3.55 A	2.78 B	3.01 B	3.49 A	

Means with different letters are significantly different at 5%

### Swelling degree

Data in Table 2 revealed a significant difference in spicklets swelling degree as affected by spathe length and picloram concentrations combined with 2iP at 0.5 mg/l or BA and 2iP both at 0.25 mg/l. The highest significant mean value appeared with spicklets removed from spathe length at 10–15 cm. Meanwhile, the lowest result was observed with the spicklets removed from spathe length (3–5 cm). Respecting to culture media, it is clear that adding picloram at 2.0 mg/l to initiation medium containing 2ip and BA both at 0.25 mg/l encouraged the highest value of swilling followed insignificantly by picloram at 1.0 mg/l with the two cytokinins. Interaction in this respect showed that, combination of 2.0 mg/l picloram +BA and 2iP at 0.25 mg/l gave the highest significant value with spicklets taken out from spathe at the length of 10–15 cm. However, the lowest significant values were recorded with spicklets removed from different spathe lengths and cultured on initiation medium added with 1.0 mg/l picloram and 0.5 mg/l 2iP.

**Table 2:** Effect of spathe length and combination between picloram, 2iP, and BA on the swelling degree of date palm explants after 8 weeks

Treatments (mg/l)	Spathe length				Mean
	3–5 cm	5–10cm	10–15cm	15–20 cm	
1.0 Picloram + 0.5 2iP	2.88 i	3.00 hi	3.66 cdef	3.22 fghi	3.19 C
1.0 Picloram + 0.25 2iP + 0.25 BA	3.44 efgh	3.55 defg	4.11 bc	4.00 bcd	3.78 AB
2.0 Picloram + 0.5 2iP	3.11 ghi	3.44 efgh	4.33 ab	3.11 ghi	3.49 BC
2.0 Picloram + 0.25 2iP + 0.25 BA	3.33 fghi	4.33 ab	4.66 a	3.88 bcde	4.05 A
Mean	3.19 B	3.58 B	4.19 A	3.55 B	

Means with different letters are significantly different at 5%

### Embryonic culture emergence percentage

Data in Table 3 and Fig. 1c showed that spicklet explants taken out from spathe lengths ranged from 10–15 cm to 15–20 cm gave the highest percentage of embryonic culture emergence (96.66 and 91.66%) in respective order. While, the percentage was extremely decreased by using spicklet explants removed from spathe length (3–5 cm). Culture medium containing 2.0 mg/l picloram + 0.25 mg/l BA + 0.25 mg/l 2iP gave the highest percentage, whereas medium added with 1.0 mg/l picloram + 0.5 mg/l 2ip recorded the lowest percentage. With respect to the interaction, data show that, the highest percentage of spicklet explants producing embryonic culture appeared in spathe length (10–15 cm) on all culture media used as percentages were ranged from 93.33 to 100%. However, the lowest percentages were recorded with spicklet explants taken out from small spathe (3–5 cm) and cultured on different media under investigation as the percentages were ranged from 17.77 to 26.66%.

**Table 3:** Effect of spathe length and combination between picloram, 2iP, and BA on the embryonic culture emergence percentage of date palm explants after 24 weeks

Treatments (mg/l)	Spathe length				Mean
	3–5 cm	5–10 cm	10–15cm	15–20 cm	
1.0 Picloram + 0.5 2iP	17.77	75.55	93.33	88.88	68.88
1.0 Picloram + 0.25 2iP + 0.25 BA	22.22	82.22	100.00	95.55	74.99
2.0 Picloram + 0.5 2iP	20.00	86.67	97.78	86.67	72.78
2.0 Picloram + 0.25 2iP + 0.25 BA	26.66	88.88	100.00	95.55	76.66
Mean	21.66	83.33	96.665	91.66	

### Globularization degree

Table 4 and Fig. 1d reflected that spiklet explants removed from spathe at length of 10–15 cm and 15–20 cm gave the highest significant mean values of embryonic culture formation (globularization) in respective order, while spiklet explants removed from spathe at length of 3–5 cm produced the lowest significant mean value. Initiation culture media containing 1.0 or 2.0 mg/l picloram + 0.25 mg/l BA + 0.25 mg/l 2iP gave the highest significant degrees of embryonic culture formation without significant differences. Interaction revealed that the highest significant degree of embryonic culture formation was achieved when spiklet explants were removed from spathe at length of 10–15 cm and cultured on MS medium enriched with 1.0 mg/l picloram and 0.25 mg/l of both 2iP and BA. Meanwhile, the lowest significant degree was recorded when explants had been taken out from small spathe (3–5 cm in length) and cultured on 1.0 mg/l picloram + 0.5 mg/l 2iP (2.22).

**Table 4:** Effect of spathe length and combination between picloram, 2iP, and BA on the globularization degree of date palm spicklet explants after 24 weeks

Treatments (mg/l)	Spathe length				Mean
	3–5 cm	5–10 cm	10–15 cm	15–20 cm	
1.0 Picloram + 0.5 2iP	2.22 h	3.22 fg	4.66 ab	3.66 def	3.44 B
1.0 Picloram + 0.25 2iP + 0.25 BA	2.66 gh	4.11 bcd	5.00 a	4.55 ab	4.08 A
2.0 Picloram + 0.5 2iP	3.11fg	3.88 cde	4.11bcd	4.22 bcd	3.83 AB
2.0 Picloram + 0.25 2iP + 0.25 BA	3.44 ef	3.66 def	4.66 ab	4.44 abc	4.05 A
Mean	2.86 C	3.72 B	4.61A	4.22 A	

Means with different letters are significantly different at 5%

### Maturation stage

Embryonic culture (0.5 g) was shifted from initiation medium and cultured on maturation medium containing mainly 0.1 mg/l picloram. ABA or 2iP at 0.1 mg/l were added to picloram-supplemented medium alone and in combination to discuss their effects on morphological responses, expressed as increase in culture fresh weight, individual, multiple, and vitrified embryo numbers after 12 weeks (6-weeks interval). It is evident from the results in Table 5 that morphological different responses of embryonic culture were mainly growth regulator-dependent. The addition of ABA and 2iP to maturation medium in the presence of picloram markedly produced the heaviest significant fresh weight (6.2 g) presented in Fig. 1e, followed by media containing picloram + 2ip, picloram alone and picloram + ABA (5.8, 4.78, and 4.4 g), in respective order. In contrast, the lowest significant value of fresh weight was noticed with control medium (growth regulator-free medium). In respect to individual embryo numbers (Fig. 1e), it is clear that the addition of ABA to picloram-containing medium achieved the highest significant number, followed significantly by media with picloram + 2iP or picloram alone, respectively, while control medium and ABA + 2iP-supplemented medium gave the lowest significant number in respective order. Combination of picloram + 2iP + ABA and combination of picloram+ 2iP encouraged the highest significant numbers of multiple embryos, respectively with significant differences. However, sharp significant decrease in multiple embryo numbers was noticed with control medium. The vitrification phenomenon clearly appeared with media containing 0.1 mg/l picloram + 0.1 mg/l 2ip (Fig. 1e), while control medium and medium containing picloram + ABA gave the lowest significant numbers of vitrified embryos in respective order.

**Table 5:** Effect of picloram, ABA, and 2iP on morphological responses of embryonic cultures after 12 weeks (6-weeks interval)

Treatments (mg/l)	Culture fresh weight (mg)	Individual embryos (***n***)	Multiple embryos (***n***)	Vitrified embryos (***n***)
Control^*^	2.30 e	15.50 c	3.20 e	6.30 c
0.1 Picloram	4.78 c	20.39 b	11.30 d	8.20 bc
0.1 Picloram + 0.1 ABA	4.40 d	27.90 a	13.80 c	6.80 c
0.1 Picloram + 0.1 2ip	5.80 b	22.50 b	16 .70 b	12.60 a
0.1 Picloram + 0.1 2ip + 0.1 ABA	6.20 a	17.50 c	22.40 a	9.10 b

Means with different letters within each column are significantly different at 5%; ^*^Control: without growth regulators

**Fig. 2:**
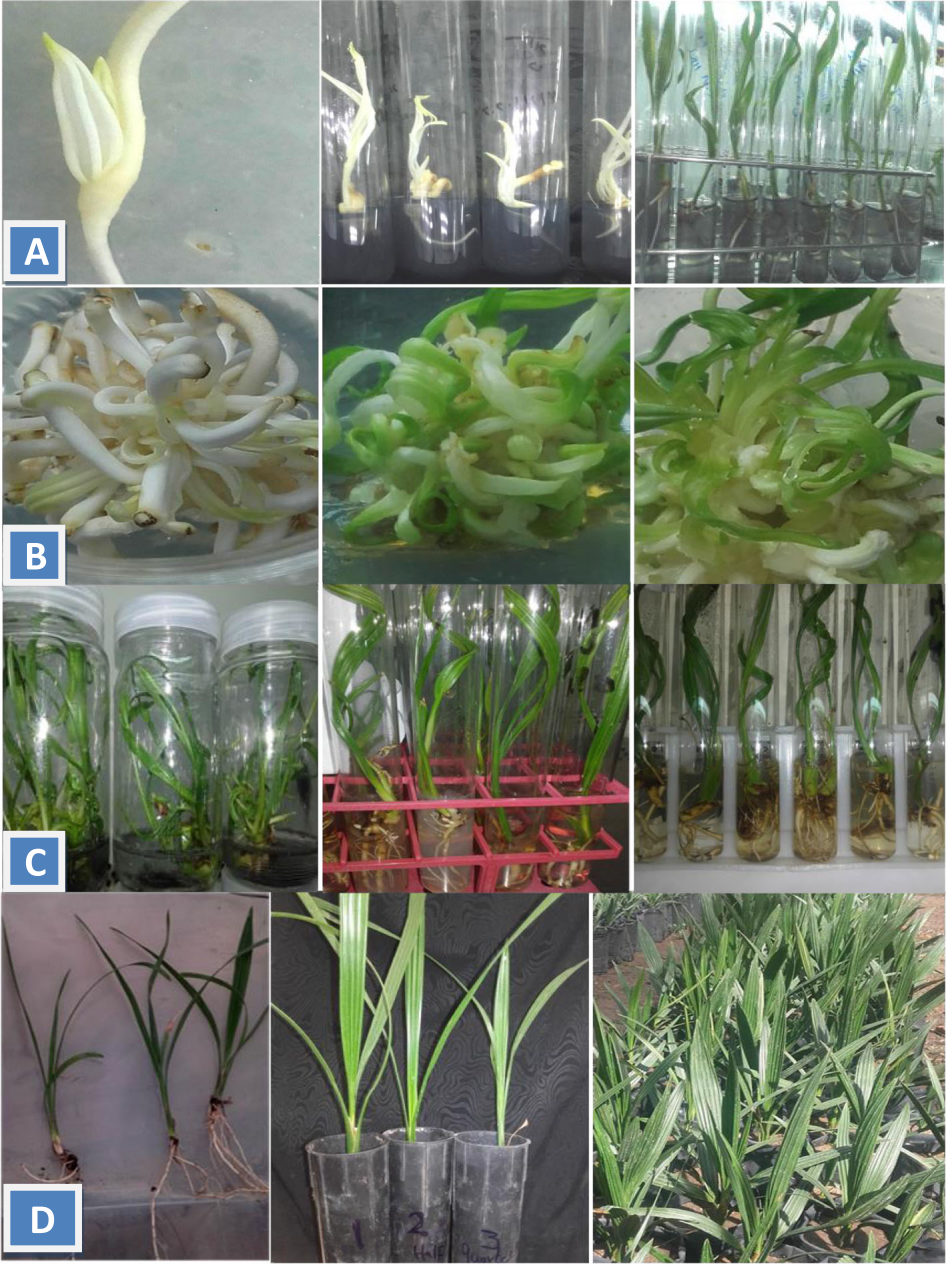
*In vitro* regeneration of Medjool cv. **a** Regeneration proceedings of individual embryos. **b** Multiplication stage as vigorous secondary embryos, healthy shoots and vitrified shoots, from left to right. **c** Elongation and two steps of rooting stage. **d** Plant growth after acclimatization; from left to right: effect of ¼, ½, and full MS strength on root growth of acclimatized plants, effect of full, ½, and ¼ MS on shoot growth of 6-month-old plants and finally 12-month-old plants ready for transferring to the field

It is worth mentioning that individual embryos need transferring to another media as previously mentioned to complete growth and development (Fig. 2a).

### Multiplication stage

Clusters of multiple embryos containing 3–4 embryos resulted from maturation stage were cultured on 3/4 MS strength media containing different cytokinins with or without activated charcoal to study their effects on multiple embryo growth and development in terms of number of secondary embryos, number of shoots, and number of vitrified shoots.

Data in Table 6 and Fig. 2b revealed that medium containing 0.25 mg/l BA generally gave the greatest significant number of secondary embryos (43.83/cluster) followed significantly by medium supplemented with a combination of BA, kinetin, and 2iP, each at 0.1 mg/l and medium with 0.25 mg/l kinetin (37.6 and 31.25/cluster, respectively), while the lowest number was noticed with control medium (8.65/cluster). In respect to activated charcoal, medium without activated charcoal achieved the highest significant number in comparison with that containing activated charcoal (32.99 and 23.84 embryo/cluster, in respective order). The interaction shows that the best result was noticed on BA-supplemented medium without activated charcoal, while the lowest significant numbers were observed with control medium with or without activated charcoal. Generally, it could be noticed that 2ip was less effective than BA and kinetin in this concern.

**Table 6:** Effect of different cytokinins and activated charcoal on secondary embryo number, shoot number, and vitrified embryos after 3 recultures

Treatments (mg/l)	Secondary embryo n.			Shoot number			Vitrified embryos ***n***		
	With AC	Without AC	Mean	With AC	Without AC	Mean	With AC	Without AC	Mean
Control (without cytokinin)	6.20 h	11.10 g	8.65 E	11.60 ef	9.42 f	10.51D	3.50 e	0.00 f	1.75 D
0.25 BA	39.00 c	48.66 a	43.83A	22.00bc	19.60 cd	20.80B	6.80 cd	7.20 c	7.00 B
0.25 Kinetin	26.00 e	36.50 c	31.25C	29.00 a	24.20 b	26.60A	4.55 de	5.60 cde	5.07 C
0.25 2ip	16.30 f	25.20 e	20.75D	17.00 d	13.50 e	15.25C	15.00 b	17.90 a	16.45 A
0.1 2ip + 0.1 kinetin + 0.1 BA	31.70 d	43.50 b	37.60B	23.00 b	18.30 d	20.65B	5.20 cde	7.50 c	6.35 BC
Mean	23.84 B	32.99 A		20.52 A	17.00 B		7.01 A	7.64 A	

Means with different letters for each parameter are significantly different at 5%

Data in Table 6 showed that the addition of kinetin to culture medium achieved the highest significant shoot number followed insignificantly by a combination of BA, kinetin, and 2iP. In contrast, control medium (without cytokinin) gave the lowest significant number. The addition of AC to the multiplication medium generally enhanced shoot number compared with medium free of AC. Interaction revealed that using culture medium containing kinetin and AC surpassed other media under investigation. However, it is evident that control media with or without AC produced the lowest significant shoot number.

Data in Table 6 showed that culture medium containing 2ip significantly increased the vitrified shoot number (16.45 vitrified shoots/cluster) presented in Fig. 2b, while only 1.75 vitrified shoot/cluster was appeared with control medium without cytokinin. In concern with activated charcoal, it is clear that no significant differences could be noticed in vitrified shoot number in relation to AC in culture medium. Interaction revealed that 2ip supplemented media with or without AC gave the highest significant numbers of vitrified shoot/cluster through 3 recultures. In contrast, control medium without cytokinin reduced significantly vitrified shoots to 0.0 in medium devoid of AC.

### Rooting and acclimatization stages

Clusters of developed shoots (4–5 shoots) which resulted from the multiplication stage were transferred to liquid culture media to obtain healthy individual shoots for rooting stage (Fig. 2c). Two steps of the rooting stage were conducted to produce healthy plantlets for acclimatization. Elongated shoots were transferred to the rooting solid medium, previously mentioned, to induce roots then to the pre-acclimatization liquid medium (Fig. 2c). Rooted plantlets with vigorous shoots and roots were successfully adapted in the greenhouse. After that, irrigation with three strengths of MS salts was conducted.

It is evident from Table 7 and Fig. 2d that, increasing MS strength which is used as a fertilizer solution for date palm plants during acclimatization stage, has a strong effect on most of parameter measurements. Furthermore, the highest significant leaf number/plant appeared with MS full strength followed insignificantly by MS half strength, while the lowest leaf number was noticed with quarter strength. Full-strength solution gave the greatest degree of leaf width followed significantly by other strengths in respective order. No significant differences in plant length could be observed among different strengths. It is generally observed from the data in Table 7 that irrigation with full-strength solution encouraged the highest significant mean values of growth vigor degree, root number, and root thickness degree. However, the lowest significant values were noticed with ¼ strength solution.

**Table 7:** Effect of Murashige and Skooge salts (MS) solution strength on growth measurements of date palm plants after 24 weeks in greenhouse conditions

MS strength	Leaf number	Leaf width (cm)	Plant length (cm)	Growth vigor	Root number	Root thickness
¼ MS	4.1 b	1.8 c	29.4 a	3.8 b	3.7 c	2.3 c
½ MS	4.5 ab	2.1 b	31. 8 a	4.2 b	4.8 b	3.8 b
Full MS	4.9 a	2.5 a	32.5 a	5.0 a	6.3 a	5.0 a

Means with different letters within each column are significantly different at 5%

## Discussion

It is clear from our results that the size of the spathe is an important factor for avoiding browning problems and low responses at initiation stage. Abul-Soad [7] studied the size of date palm spikelets not spathes in a Pakistani cultivar ‘Aseel’ at 5–15, 15–20, 25–30, and 40–45 cm cultured for indirect embryogenesis and found that the smallest size showed browning and low response while the two followed sizes gave pro-embryo cultures and the bigger size gave unfriable callus. Our result showed that the moderate size is preferable than the smallest or biggest size of spathe (5–10 and 10–15 cm) especially the third size (10–15) that gave the best swilling, response percent, and globularization.

Although the auxin 2, 4-dichlorophenoxy acetic acid (2, 4-D) has been frequently used to induce somatic embryogenesis in date palm [22] but higher concentrations may induce somaclonal variation [23]. Therefore, using other auxins at low concentrations to induce somatic embryogenesis in date palm would be of great interest. Picloram could be an alternative auxin as it was used later for induction of peach palm [32], African oil palm [33], areca nut palm [34], and macauba palm [35] somatic embryos. In date palm, picloram has been used to induce callus culture and somatic embryogenesis by using a higher concentration range; from 10.8 mg/l (for shoot tip) to 50 mg/l (for leave explants) [36, 25, respectively]. The current study targeted inducing direct embryogenesis from palm inflorescence using low concentrations of picloram (1.0 and 2.0 mg/l with 2ip or 2ip + BA). Results clearly showed that combinations of picloram with 2ip and BA gave the highest response for embryonic culture and globularization. Similarly, inducing somatic embryogenesis from adventitious bud segments of Mejhoul shoot tips was encouraged by different concentrations of picloram [36]. The surpassed medium for somatic embryogenesis induction (70%) was MS medium supplemented with 10.8 mg/l picloram and 1.0 mg/l 2iP. The optimal somatic embryogenesis in *Picea abies* was obtained with picloram editing to the initiation medium. Moreover, addition of BA to picloram-enriched medium enhanced the response [37].

Resulted embryonic cultures (in maturation stage) were transferred to medium with lower concentration of picloram (0.1 mg/l) alone, with 2ip, ABA or both of them to induce a matured embryo. A higher response rate (culture fresh weight and multiple embryos) was induced by the combination of picloram and both 2ip and ABA at 0.1 mg/l. Decrement of the picloram concentration also stimulated the maturation process resulting ultimately in the germination of somatic embryos that exhibited bipolar development of rattan species [38]. The presence of abscisic acid (ABA) seemed to be beneficial for obtaining healthy embryos (without vitrification). Furthermore, ABA increased the number of individual embryos with the presence of picloram as well as multiple embryo numbers when 2ip is combined with both of them. Similarly, Ibrahim et al*.* [39] stated that ABA induced numerous embryos and stimulated matured embryos of date palm cv. Sakkoty. A few protocols could describe the use of ABA to improve embryo maturation [40, 41]. Zouine et al. [42] reported that ABA at the concentration of 10^−5^ M increased sugar and total protein accumulation in somatic embryos of date palm cvs. Bousthami Noir and Jihel.

In previous studies, many factors have been associated with somatic embryo germination. Ibrahim et al. [43] reported that genotype and desiccation influence embryo germination in date palm. Moreover, the size of somatic embryos [44] and the texture of the culture medium [45] were also reported to influence somatic embryo germination in date palm. Zouine et al. [42] assured that activated charcoal promotes somatic embryo germination in date palm cvs. Bousthami Noir and Jihel, and these are in harmony with our results as AC increased germination (as shoot number).

Many others tried to understand the role of auxins or auxin congeners in embryogenesis. They suggested that auxins, when added to the culture medium, interfere with explant endogenous hormones and affect their levels [46]. This might cause stress conditions that encourage the embryogenic transition in somatic cells [47]. Every auxin may interfere in a different way due to the involvement of different signal transductions [48]. Otherwise, the development of genetic approaches to the study of plant hormone signaling led to the discovery that auxin acts by promoting degradation of transcriptional repressors called ‘Aux/IAA’ proteins. They have been shown to act like molecular glue binding to its TIR1 receptor and promote ubiquitin-dependent degradation of ‘Aux/IAA’ repressor proteins, activating the auxin response elements [49]. Naturally occurring auxin (IAA) and synthesized auxin congeners (i.e., NAA and 2, 4-D) showed the same activity. It is thought that auxin congeners also have a dual role during the induction of somatic embryogenesis, one related to auxin signalling and the other to a stress component [47] that also changes the endogenous content of auxins [50].

During the acclimatization stage of date palm plants, full MS strength used as a fertilizer solution has a strong effect on most of parameter measurements in this investigation. A later protocol for large-scale production of micropropagated date palm plantlets was achieved. Hoagland solution enhanced plant growth and developmental parameters including plant height, leaf width, stem base diameter, chlorophyll A and B, carotenoids, and total indoles [51].

## Conclusions

This research provides an advanced regeneration system for large-scale production of date palm from immature inflorescences of Medjool cv. through direct somatic embryo. We recommend using the moderate size of spathe (10–15 cm) and culturing on MS medium enriched with 1.0 mg/l picloram for direct embryogenesis induction. ABA with decrement concentration of picloram was vital for individual embryo figuration, while 2ip with picloram was necessary for multiple embryo consistencies. The research reported here opens up the prospects of using picloram with different growth regulators for rapid micropropagation of date palm.

## Data Availability

All data generated or analyzed during this study are included in this published article.

## Abbreviations

NAA: Naphthaleneacetic acid
NOA: Naphtoxyacetic acid
BA: Benzyleadenine
2ip: N6-(2-Isopentenyl) adenine
Kin: Kinetin
HgCl_2_: Mercuric chloride
IAA: Indole acetic acid
IBA: Indole-3-butyric acid
ABA: Abscisic acid
MS: Modified medium described by Murashige and Skoog (1962)
PVP: Polyvinyl pyrrolidone
AC: Activated charcoal
Pbz: Paclobutrazol

## References

[1] Johnson DV, Al-Khayri JM, Jain SM, Al-Khayri JM, Jain SM, Johnson DV (2015). Introduction: date production status and prospects in Asia and Europe. Date palm genetic resources and utilization.

[2] Tisserat B (1983). Tissue culture of date palm—a new method to propagate an ancient crop and a short discussion of the California date industry. Principes.

[3] Jain SM (2012). *In vitro* mutagenesis for improving date palm (*Phoenix dactylifera* L.). Emir J Food Agric.

[4] Hassan MM, Taha RA, Ibrahim IA (2016). *In vitro* conservation of date palm embryos under slow-growth conditions with osmotic agent and abscisic acid. Inter J PharmTech Res.

[5] Hassan MM, Abd-El Kareim AHI, Hussein FA, Shams El-Din IM (2017). IBA and TDZ induced plant regeneration of date palm through immature female inflorescence culture. Inter J Adv Agric Sci Technol.

[6] Zayed EMM, Zein El-Din AFM, Manaf HH, Abdelbar OH (2016). Floral reversion of mature inflorescences of date palm *in vitro*. Ann Agric Sci.

[7] Abul-Soad A (2012). Influence of inflorescence explant age and 2,4-D incubation period on somatic embryogenesis of date palm. Emirates J Food Agri.

[8] Gadalla EG, Hassan MM, Al-Sharabasy SF (2015). Effect of growth regulators on somatic embryogenesis of date palm inflorescence cv.

[9] Stino RG, El-Kosary S, Hassan MM, Kinawy AA (2015). Direct embryogenesis from inflorescences culture of Sewy date palm (*Phoenix dactylifera* L.). J Biol Chem Environ Sci.

[10] Zaid A, Al Kaabi HH, El Korchi B (2007). Large scale *in vitro* propagation of a rare and unique male date palm (*Phoenix dactylifera* L.) using inflorescences technique. Acta Horti.

[11] Kriaa W, Sghaier B, Masmoudi F, Benjemaa R, Drira N (2012). The date palm (*Phoenix dactylifera* L.) micropropagation using completely mature female flowers. C R Biol.

[12] Abahmane L (2013). Recent achievements in date palm (*Phoenix dactylifera* L.) micropropagation from inflorescence tissues. Emir J Food Agri.

[13] Taha RA, Al-Khayri JM (2017). Enhanced indirect somatic embryogenesis of date palm using low levels of seawater. Date palm biotechnology protocols V1: tissue culture applications, methods Mol biol.

[14] Fki L, Masmoudi R, Drira N, Rival A (2003). An optimized protocol for plant regeneration from embryogenic suspension cultures of date palm, *Phoenix dactylifera* L., cv. Deglet Nour. Plant Cell Rep.

[15] Al-Khayri JM, Jain SM, Gupta PK (2005). Date palm *Phoenix dactylifera* L. Protocols of somatic embryogenesis in Woody plants.

[16] Hassan MM, Taha RA (2012). Callogenesis, somatic embryogenesis and regeneration of date palm *Phoenix dactylifera* L. cultivars affected by carbohydrate sources. Inter J Agri Res.

[17] Taha RA, Hassan MM, Ibrahim EA, Abo-Bakr NH, Shaaban EA (2016). Carbon nanotubes impact on date palm *in vitro* cultures. PCTOC.

[18] George EF, Hall MA, De Klerk GJK (2008). Plant propagation by tissue culture.

[19] El Hadrami A, Daayf F, Elshibli S, Jain SM, El Hadrami I, Jain SM, Al-Khayri JM, Johnson DV (2011). Somaclonal variation in date palm. Date palm biotechnology.

[20] Rademacher W (2000). Growth retardants: effects on gibberellin biosynthesis and other metabolic pathways. Annu Rev Plant Physiol Plant Mol Biol.

[21] Chen JT, Chang WC (2003). Effects of GA3, ancymidol, cycocel and paclobutrazol on direct somatic embryogenesis of *Oncidium in vitro*. PCTOC.

[22] Al-Khayri JM (2010). Somatic embryogenesis of date palm (*Phoenix dactylifera* L.) improved by coconut water. Biotechnol.

[23] Fki L, Masmoudi R, Kriaâ W, Mahjoub A, Sghaier B, Mzid R, Mliki A, Rival A, Drira N, Jain SM, Al-Khayri JM, Johnson DV (2011). Date palm micropropagation via somatic embryogenesis. Date palm biotechnology.

[24] Abahmane L (2010). Micropropagation of date palm (*Phoenix dactylifera* L) selected genotypes from inflorescence tissues by using somatic embryogenesis technique. Acta Hortic.

[25] Othmani A, Bayoudh C, Drira N, Trifi M (2009). *In vitro* cloning of date palm *Phoenix dactylifera* L., cv. Deglet Bey by using embryogenic suspension and temporary immersion bioreactor (TIB). Biotechnol Biotechnol Equip.

[26] Khierallah HSM, Al-Hamdany MHS, Abdulkareem AA, Saleh FF (2015). Influence of sucrose and paclobutrazol on callus growth and somatic embryogenesis in date palm cv. Bream. Int J Curr Res Aca Rev.

[27] Mazri MA, Belkoura I, Meziani R, Mokhless B, Nour S (2017). Somatic embryogenesis from bud and leaf explants of date palm (*Phoenix dactylifera* L.) cv. Najda. 3. Biotech.

[28] Murashige T, Skoog F (1962). A revised medium for rapid growth and bioassays with tobacco tissue culture. Physiol Plant.

[29] Hassan MM, Ibrahim IA, Fathy NM, Ebrahim MK, Komor E (2014). Protocol for micropropagated date palm acclimatization: effect of micropropagated plantlet type, soil composition, and acclimatization season. Inter J Fruit Sci.

[30] Pottino BG (1981). Methods in plant tissue culture.

[31] Snedecor WB, Cochran GW (1989). Statistical methods.

[32] Steinmacher DA, Clement CR, Guerra MP (2007). Somatic embryogenesis from immature peach palm inflorescence explants: towards development of an efficient protocol. PCTOC.

[33] Teixeira JB, Söndahl MR, Nakamura T, Kirby EG (1995). Establishment of oil palm cell suspensions and plant regeneration. PCTOC.

[34] Karun A, Siril EA, Radha E, Parthasarathy VA (2004). Somatic embryogenesis and plantlet regeneration from leaf and inflorescence explants of areca nut (*Areca catechu* L.). Curr Sci.

[35] Moura EF, Ventrella MC, Motoike SY, de Sa AQ, Carvalho M, Manfio CE (2008). Histological study of somatic embryogenesis induction on zygotic embryos of macaw palm (*Acrocomia aculeata* (Jacq.) Lodd. Ex Martius). PCTOC.

[36] Mazri MA, Meziani R, Belkoura I, Mokhless B, Nour SA (2018). Combined pathway of organogenesis and somatic embryogenesis for an efficient large-scale propagation in date palm (*Phoenix dactylifera* L.) cv. Mejhoul. 3 Biotech.

[37] Hazubska-Przybył T, Ratajczak E, Obarska A, Pers-Kamczyc E (2020). Different roles of auxins in somatic embryogenesis efficiency in two Picea species. Int J Mol Sci.

[38] Goh DKS, Bon MC, Aliotti F, Escoute J, Ferrière N, Monteuuis O (2001). *In vitro* somatic embryogenesis in two major rattan species: *Calamus merrillii* and *Calamus subinermis*. In Vitro Cell Dev Biol Plant.

[39] Ibrahim IA, Hassan MM, Taha RA (2011). Morphological studies on date palm micropropagation as a response to growth retardants application.

[40] Teixeira JB, Söndahl MR, Kirby EG (1994). Somatic embryogenesis from immature inflorescences of oil palm. Plant Cell Rep.

[41] Jayanthi M, Susanthi B, Murali Mohan N, Mandal PK (2015). *In vitro* somatic embryogenesis and plantlet regeneration from immature male inflorescence of adult dura and tenera palms of *Elaeis guineensis* (Jacq.). Springerplus.

[42] Zouine J, El Bellaj M, Meddich A, Verdeil JL, El Hadrami I (2005). Proliferation and germination of somatic embryos from embryogenic suspension cultures in *Phoenix dactylifera*. PCTOC.

[43] Ibrahim IA, Hassan MM, Taha RA (2012). Partial desiccation improves plant regeneration of date palm *in vitro* cultures. Wudpecker J Agri Res.

[44] Al-Khayri JM, Al-Bahrany AM (2012). Effect of abscisic acid and polyethylene glycol on the synchronization of somatic embryo development in date palm (*Phoenix dactylifera* L.). Biotechnol.

[45] Boufis N, Khelifi-Slaoui M, Djillali Z, Zaoui D, Morsli A, Bernards MA, Makhzum A, Khelifi L (2014). Effects of growth regulators and types of culture media on somatic embryogenesis in date palm (*Phoenix dactylifera* L. cv. Degla Beida). Sci Hortic.

[46] Gaspar T, Kevers C, Penel C, Greppin H, Reid DM, Thorpe TA (1996). Plant hormones and plant growth regulators in plant tissue culture. In Vitro Cell Dev Biol Plant.

[47] Feher A, Mujib A, Samaj J (2005). Why somatic plant cells start to form embryos?. Somatic embryogenesis.

[48] Pacurar DI, Perrone I, Bellini C (2014). Auxin is a central player in the hormone cross-talks that control adventitious rooting. Physiol Plant.

[49] Tan X, Calderon-Villalobos LI, Sharon M, Zheng C, Robinson CV, Estelle M, Zheng N (2007). Mechanism of auxin perception by the TIR1 ubiquitin ligase. Nature.

[50] Jiménez V (2005). Involvement of plant hormones and plant growth regulators on *in vitro* somatic embryogenesis. Plant Growth Regul.

[51] Hassan MM, Al-Khayri JM (2017). Improvement of *in vitro* date palm plantlet acclimatization rate with kinetin and Hoagland solution. Date palm biotechnology protocols volume 1: tissue culture applications, methods Mol biol, 1637.

